# Impact of SARS-CoV-2 Omicron on Rapid Antigen Testing Developed for Early-Pandemic SARS-CoV-2 Variants

**DOI:** 10.1128/spectrum.02006-22

**Published:** 2022-08-09

**Authors:** Karoline Leuzinger, Tim Roloff, Adrian Egli, Hans H. Hirsch

**Affiliations:** a Clinical Virology, Laboratory Medicine, University Hospital Basel, Basel, Switzerland; b Transplantation & Clinical Virology, Department Biomedicine, University of Basel, Basel, Switzerland; c Applied Microbiology Research, Laboratory Medicine, Department Biomedicine, University of Basel, Basel, Switzerland; d Clinical Bacteriology and Mycology, Laboratory Medicine, University Hospital Basel, Basel, Switzerland; e Infectious Diseases & Hospital Epidemiology, University Hospital Basel, Basel, Switzerland; NIAID

**Keywords:** severe acute respiratory syndrome coronavirus 2, SARS-CoV-2, Omicron, variant, COVID-19, BA.2, BA.5, rapid antigen tests, RAT

## Abstract

Rapid antigen tests (RATs) are widely used for point-of-care or self-testing to identify SARS-CoV-2 (SCoV2), but currently circulating Omicron variants may impair detection. In this study, we prospectively evaluated the Roche-SARS-CoV-2-Antigen and Acon-FlowFlex-SARS-CoV-2-Antigen in 150 consecutively collected nasopharyngeal patient swabs (50 SCoV2 RNA undetectable; 100 SCoV2 Omicron BA.1). Omicron BA.1 results were compared to 92 Ct-matched early-pandemic SCoV2 variants (B.1.160 and B.1.177), to 100 Omicron BA.2 positive and to 100 Omicron BA.5 positive samples. For Omicron BA.1, Roche-SARS-CoV-2-Antigen detected 87% of samples having Ct-values <29 reflecting 3.6% lower rates compared to B.1.160 and B.1.177. Acon-FlowFlex-SARS-CoV-2-Antigen was less affected and detected 90% of Omicron BA.1 with Ct-values <29. Omicron BA.2 and BA.5 detection rates were significantly reduced by 20% and 10%, respectively, for the Roche-SARS-CoV-2-Antigen in samples with Ct-values <29 but remained similar for Acon-FlowFlex-SARS-CoV-2-Antigen. RATs need to be continuously evaluated as new SCoV2-variants emerge. Spreading of Omicron-BA.2, and the recently emerged Omicron BA.5 variant, may not only result from escape from postvaccine or postinfection immunity, but also from false-negative RATs misguiding point-of-care and self-testing decisions at times of restricted molecular testing.

**IMPORTANCE** Antigen tests are widely used for rapid identification of SCoV2-positive cases and their increased risk of transmission. At present, there are several FDA- and CE-cleared tests available in North America and Europe. However, their diagnostic performance has been evaluated with early-pandemic variants. This study provides evidence that variation within the nucleocapsid protein as seen in recently emerged and now globally spreading Omicron BA.2 and BA.5 variants significantly impairs detection rates of widely used antigen tests. Consequently, antigen tests need to be reevaluated when new pandemic SCoV2 variants emerge and start to predominate globally.

## INTRODUCTION

Rapid antigen tests (RAT) have become an important corner stone in SARS-CoV-2 (SCoV2) diagnostics for rapid and low cost SCoV2 detection in respiratory fluids, facilitating screening at hospitals, schools, offices and at home. Although molecular assays using nucleic acid testing (NAT) are preferred for symptomatic patients with risk factors or needing hospital admission and treatment (https://www.covid19.admin.ch/de/overview), RATs permit point-of-care as well as self-testing and facilitate timely decisions regarding infection control measures and self-isolation to curtail transmission of SCoV2. Indeed, for high SCoV2 loads with cycling threshold (Ct) values of less than 29, RATs were reported to have SCoV2 detection of >90% (1). However, sensitivity of RATs may be impaired for novel SCoV2 variants, such as the now globally dominating Omicron variants (https://nextstrain.org/ncov/gisaid/global). Importantly, Omicron strains harbor a number of mutations in the otherwise rather conserved nucleocapsid protein (BA.1, BA.2 and BA.5: P13L, Δ31-33, R203K, G204R; and for BA.2 and BA.5 additionally S413R; https://covariants.org/variants/21K.Omicron) that may potentially impair antigen detection rates. In this prospective clinical validation study, we analyzed nasopharyngeal swabs from patients with and without NAT confirmed SCoV2 infection and prospectively assessed the diagnostic performance of the Roche-SARS-CoV-2-Antigen that has received emergency use authorization (EUA) by the food and drug administration (FDA) and is widely employed in Northern American and European health care. Results were compared to Ct-matched early-pandemic SCoV2 variants (B.1.160 and B.1.177) and the Acon-FlowFlex-SARS-CoV-2-Antigen that is widely available in retail and pharmacy stores.

## RESULTS

### Prospective evaluation of the Roche-SARS-CoV-2-Antigen.

From January 25 to February 28^,^ 2022, SCoV2 specific NAT testing was done using the cobas SARS-CoV-2 test on the cobas6800 platform from 150 nasopharyngeal patient swabs and analyzed in parallel with the Roche-SARS-CoV-2-Antigen. All 50 SCoV2 RNA negative samples were also non-reactive in the Roche-SARS-CoV-2-Antigen (specificity of 100%; 95%CI: 92.9% to 100%). Of 100 Omicron BA.1 positive samples, 80 (80%) were detected by the *Roche-*SARS-CoV-2-Antigen. Omicron BA.1 positive samples with low SCoV2 RNA loads requiring Ct-values of >30 (*n* = 8) were non-reactive in the Roche-SARS-CoV-2-Antigen. For samples with Ct-values <29 (approximately 10,000 copies/mL or 4 log_10_ c/mL), sensitivity was 87% (95%CI: 78.3% to 93.1%) with a negative predictive value (NPV) of 98.6% at current local SCoV2 prevalence of <10% (https://www.covid19.admin.ch/de/overview). The Roche-SARS-CoV-2-Antigen sensitivity significantly increased with SCoV2 loads, having a sensitivity of 94% (95%CI: 86.2% to 98.0%) in samples with Ct-values <26 (approximately 100’000 c/mL or 5 log_10_ c/mL), and 100% (95%CI: 93.4% to 100%) in samples with Ct-values <23 (approximately 1 million c/mL or 6 log_10_ c/mL).

### SCoV2 early-pandemic and Omicron variant detection by the Roche-SARS-CoV-2-Antigen.

**FIG 1:**
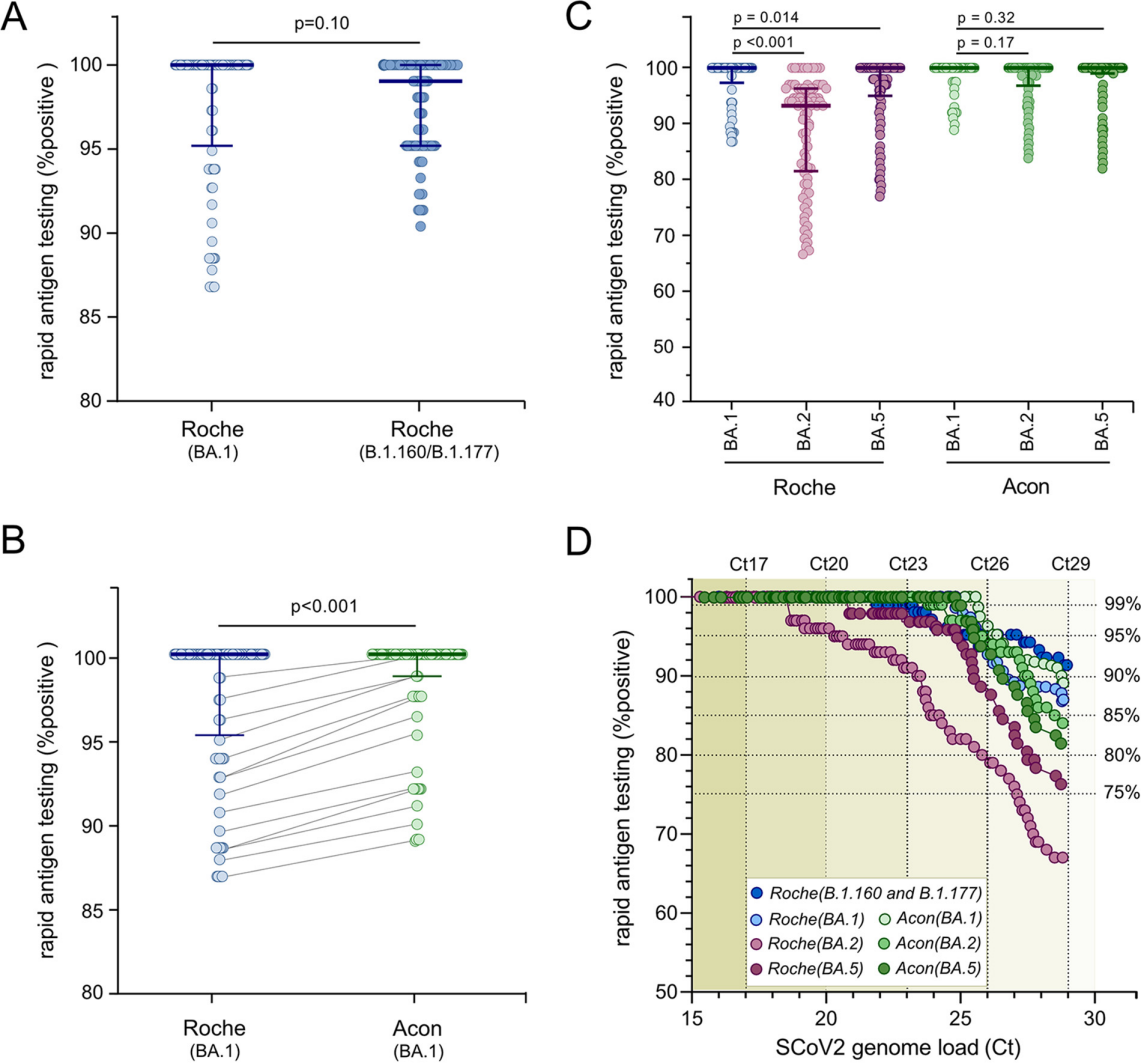
Comparison of the cumulated sensitivity for Omicron detection with the Roche-SARS-CoV-2 and the Acon-FlowFlex-SARS-CoV-2 rapid antigen tests. Cumulative sensitivity of the Roche-SARS-CoV-2 and the Acon-FlowFlex-SARS-CoV-2 RATs for Omicron BA.1 detection were compared in Ct-matched UTM samples with early-pandemic SCoV2 variants (B.1.160 and B.1.177; *n* = 92) (1), Omicron BA.2 and Omicron BA.5 (*n* = 100). SCoV2 loads were determined using the cobas SARS-CoV-2 test on the cobas6800 platform (Roche). A. Cumulated sensitivity of the Roche-SARS-CoV-2 (BA.1) and the Roche-SARS-CoV-2(B.1.160 and B.1.177*)* (*n* = 92; median, 25th and 75th percentile; Mann-Whitney-U test). B. Cumulated sensitivity of the Roche-SARS-CoV-2-Antigen (BA.1) and the Acon-FlowFlex-SARS-CoV-2 (BA.1) (*n* = 92; median, 25th and 75th percentile; Mann-Whitney-U test). C. Cumulated sensitivity of the Roche-SARS-CoV-2 and the Acon-FlowFlex-SARS-CoV-2 for Omicron BA.1, Omicron BA.2 and Omicron BA.5 (n = 100; median, 25th and 75th percentile; Mann-Whitney-U test). D. Receiver operating characteristic analysis (ROC) curves for the Roche-SARS-CoV-2 and the Acon-FlowFlex-SARS-CoV-2 for the different SCoV2 variants stratified by SCoV2 RNA loads.

To evaluate the diagnostic performance of the Roche-SARS-CoV-2 RAT for Omicron BA.1 and earlier SCoV2 variants (B.1.160 and B.1.177), 92 Ct-matched nasopharyngeal patient swabs from a previous study (1) were identified and compared to the current data set. By comparison, the Roche-SARS-CoV-2-Antigen rates for B.1.160 and B.1.177 were higher by 3.5%, 2.5% and 0.0% in samples with Ct-values of 29, 26 and 23. Thus, 90% detection rates in patient swabs required Ct-values <26 for Omicron BA.1, compared to Ct-values <29 for early-pandemic variants (Fig. 1A and D), corresponding to a 10-fold higher SCoV2 RNA load.

### Comparison of the Roche-SARS-CoV-2-Antigen and Acon-FlowFlex-SARS-CoV-2.

To examine the performance of another independent RAT, the 150 nasopharyngeal patient swabs were additionally analyzed by the Acon-FlowFlex-SARS-CoV-2. Of 100 Omicron BA.1 positive samples, 79 (79%) were concordant-positive, 17 (17%) concordant-negative, and 4 (4%) discordant results. The four discordant samples were Acon-FlowFlex-SARS-CoV-2-positive/Roche-SARS-CoV-2-negative and showed Ct-values >25. Overall, Acon-FlowFlex-SARS-CoV-2 antigen positivity rates were significantly increased compared to Roche-SARS-CoV-2 (*P < *0.001; Fig. 1B), with >90% Omicron BA.1 detection in clinical patient swabs with Ct-values <29, compared to 87% for Roche-SARS-CoV-2-Antigen (Fig. 1D).

### Comparison of Omicron BA.1, BA.2, and BA.5 detection.

Lastly, we compared Omicron BA.1, Omicron BA.2 and Omicron BA.5 variant detection with the Roche-SARS-CoV-2 and Acon-FlowFlex-SARS-CoV-2 RATs. Omicron BA.2 and Omicron BA.5 positive nasopharyngeal patient swabs were analyzed in parallel by the Roche-SARS-CoV-2 and Acon-FlowFlex-SARS-CoV-2 RATs, and results were compared to 100 Ct-value matched BA.1 positive patient swabs (Fig. 1C). Compared to Omicron BA.1, Omicron BA.2 significantly reduced (*P* < 0.01) detection rates by 20% for the Roche-SARS-CoV-2 in samples with Ct-values <29, while it did not affect Acon-FlowFlex-SARS-CoV-2 sensitivity. Similarly, Omicron BA.5 detection rates were significantly reduced by 10% for the Roche-SARS-CoV-2 RAT in samples with Ct-values <29 (*P* = 0.014), but remained similar for Acon-FlowFlex-SARS-CoV-*2* (Fig. 1C and D).

## DISCUSSION

Rapid antigen tests are widely used for point-of-care or for self-testing to readily identify SCoV2 infection for epidemiologic and therapeutic purposes. In this study, we prospectively assessed the diagnostic performance of the Roche-SARS-CoV-2-Antigen that is widely employed in Northern American and European health care, and the Acon-FlowFlex-SARS-CoV-2-Antigen that is widely available in retail and pharmacy stores. Our study has three major findings:

First, Roche-SARS-CoV-2-Antigen and Acon-FlowFlex-SARS-CoV-2-Antigen RATs remain effective in detecting the SCoV2 Omicron BA.1 variant in clinical nasopharyngeal swab samples with high viral loads despite extensive mutations in the nucleocapsid protein. Thus, timely decision-making remains reliable for persons with highly infectious Omicron BA.1 titers as seen in persons with little or no SCoV2-specific immunity and persons presenting early in the natural course of infection (2).

Second, the sensitivity of the Roche-SARS-CoV-2-Antigen is reduced for Omicron BA.1 compared to the early-pandemic SCoV2 variants B.1.160 and B.1.177 at lower SCoV2 loads (Ct-values >26), corresponding to SCoV2 loads of approximately 100’000 copies/mL. Thus, in vaccinated persons and those presenting late in the natural course of infection, RATs like the Roche-SARS-CoV-2-Antigen may not deliver reliable diagnostics of SCoV2/CoVID19 and a molecular test is preferred. Indeed, SCoV2 loads typically decrease by 3 log_10_ c/mL over a period of 10 days in unvaccinated patients even in the absence of effective antivirals or monoclonal antibodies (3). Conversely, vaccinated individuals have been reported to clear SCoV2 faster than unvaccinated patients (4), thereby reducing the SCoV2 transmission risk as Ct-values increase from 26 to 29 (5).

Third, not all RATs behaved similarly as the Acon-FlowFlex-SARS-CoV-2-Antigen showed a slightly better detection rate in Omicron positive samples with lower SCoV2 loads (Ct-values of 26 to 29). This trend to better detection became highly significant for the emerging Omicron BA.2 and BA.5 variants.

Finally, unlike the Acon-FlowFlex-SARS-CoV-2-Antigen, the Roche-SARS-CoV-2-Antigen was dramatically impaired in detecting the Omicron BA.2 and to a lesser degree the Omicron BA.5 variant. As the diagnostic performance of the majority of commercially available RATs is based on the use of monoclonal antibodies against specific domains of the viral nucleocapsid protein, already single amino acid exchanges, such as S413R, may significantly decrease target binding and subsequent detection rates. This poses an important diagnostic issue as Omicron BA.5 is more transmissible than BA.1, and thus have become more prevalent worldwide.

In conclusion, Roche-SARS-CoV-2-Antigen remains effective to detect Omicron BA.1 in patient samples with high SCoV2 loads, but detection of Omicron BA.2 and Omicron BA.5 is impaired. The Acon-FlowFlex-SARS-CoV-2-Antigen showed a better detection rate underlining the fact that there are substantial differences between commercially available RATs. We conclude that RATs need to be continuously evaluated as new pandemic SCoV2 variants emerge. We suggest that public health authorities should provide guidance how to appropriately define relicensing requirements. Importantly, spreading of the new Omicron BA.5 variant may not only result from postvaccine or postinfection immune escape, but also from false-negative rapid antigen testing misguiding point-of-care and self-testing decisions.

## MATERIALS AND METHODS

### Clinical specimens and SCoV2 NAT testing.

Nasopharyngeal sites were swabbed from 150 consecutive outpatients presenting to the CoVID-19 Triage and Test Center at UHB for SCoV2 specific NAT testing from January 25^th^ to February 28^th^ 2022 (6). Swabs were collected in universal transport medium (UTM; Copan; Brescia, Italy), and analyzed using the cobas SARS-CoV-2 test on the cobas6800 platform (Roche) as described (3). Cobas SARS-CoV-2 Target 1 (ORF1a/b) Ct-values were used to assess RAT performance. Omicron BA.1 was confirmed in the 100 SCoV2-positive nasopharyngeal swabs. As Omicron BA.2 and BA.5 harbor the additional nucleocapsid mutation S413R, we obtained 100 Omicron BA.2 and 100 Omicron BA.5 positive nasopharyngeal swabs from consecutive outpatients at UHB from March 8 to July 15, 2022.

### SCoV2 Omicron identification.

From all SCoV2 RNA positive samples, identification, and differentiation of Omicron BA.1, BA.2 and BA.5 was performed using three SCoV2 variant specific NATs, following the NAT cycling protocol described in (7) (for primers and probes see Table 1). The only adaptation in NAT cycling conditions was annealing and extension for 60 s at 50°C (E484A and Del96/70-NATs) and 56°C (N50Y-NAT), respectively.

**TABLE 1:** Forward primer, reverse primer, and probes of the Del96/70, E484A and N501Y NATs

Mutation	Primer/probe	Sequence (5′→3′)	Position^*a*^
Del69/70	Forward primer	CTTACCTTTCTTTTCC	21727–21742
	Reverse primer	GGTTATCAAACCTCTTAG	21806–21789
	Probe wild type	GGTTCCATGCTATACA	21753–21768
	Probe mutant-version1	GTTCCATGCTATCTCT	21754–21768
	Probe mutant-version 2	GGTTCCATGTTATCTCT	21753–21768
E484A	Forward primer	ACTGAAATCTATCAGG	22970–22985
	Reverse primer- version1	TGTAAAGGAAAGTAAC	23040–23025
	Reverse primer- version2	CGTAAAGGAAAGTAAC	23040–23025
	Probe wild type	TGTAATGGTGTTGAA	23000–23014
	Probe mutant	TTGTAATGGTGTTGCA	23001–23014
N501Y	Forward primer- version1	AAGGTTTTAATTGTTACTTTCC	23013–23034
	Forward primer- version2	CAGGTTTTAATTGTTACTTTCC	23013–23034
	Reverse primer	GAAGTTCAAAAGAAAGTACTAC	23114–23093
	Probe wild type	TTCCAACCCACTAATGG	23051–23067
	Probe mutant-version1	TTCCAACCCACTTATGG	23051–23067
	Probe mutant-version2	TTCCGACCCACTTATGG	23051–23067

a Positions according to SARS-CoV-2 isolate Wuhan-Hu-1 (acc. no. NC_045512.2).

### SCoV2 antigen testing.

For the prospective evaluation of the Roche-SARS-CoV-2-Antigen and Acon-FlowFlex-SARS-CoV-2-Antigen RATs, we assessed 100 consecutive SCoV2 Omicron BA.1 positive and 50 consecutive SCoV2 RNA negative, as well as 100 Omicron BA.2 and 100 Omicron BA.5 positive UTM samples. All samples were stored at 4°C and used within 24 h from collection. RATs were performed according to the manufacturers' instructions. Briefly, UTM samples were brought to room-temperature and 300 μL UTM was added to the extraction buffer (1:1 ratio). Three drops were added to the device and read-out was performed after 20 Min as described (1).

### Statistical methods.

Sensitivity and specificity of each RAT was calculated according to results from SCoV2-specific NAT testing. Receiver operating characteristic analysis (ROC) was done using the sensitivities of each RAT stratified by SCoV2-RNA-loads. All statistical data analysis was done in R (version3.6.1; https://cran.r-project.org), using Prism (version8; GraphPad Software, CA, USA) for data visualization. Mann–Whitney-U test was used as indicated. To evaluate the diagnostic performance of the Roche-SARS-CoV-2-Antigen RAT for Omicron BA.1 and earlier SCoV2 variants (B.1.160 and B.1.177) (1), Ct-matched UTM-samples were identified and compared to the current data set.
